# Vaccination of 1-day-old pigs with a porcine reproductive and respiratory syndrome virus (PRRSV) modified live attenuated virus vaccine is able to overcome maternal immunity

**DOI:** 10.1186/s40813-018-0101-x

**Published:** 2018-11-15

**Authors:** Monica Balasch, Maria Fort, Lucas P. Taylor, Jay G. Calvert

**Affiliations:** 1 Zoetis Manufacturing & Research Spain S.L., Ctra. Camprodon s/n, Finca La Riba, 17813, Girona, Vall de Bianya Spain; 2 Zoetis Inc., 333 Portage St, Kalamazoo, MI 49007 USA

**Keywords:** Porcine reproductive and respiratory syndrome, Modified live vaccine, Maternal-derived immunity

## Abstract

**Background:**

The objective of the study was to evaluate the influence of maternally derived antibodies (MDA) on the efficacy of a PRRSV-1 based attenuated vaccine, when administered in 1 day-old piglets by the intramuscular route. The protective immunity of the modified live virus vaccine was evaluated in pigs born from seropositive sows, vaccinated at 1 day of age, upon inoculation with a PRRSV-1 isolate. The animals were challenged when the levels of MDAs detected by seroneutralization test (SNT) in the non-vaccinated control group became undetectable (10 weeks after vaccination).

**Results:**

A protective effect of vaccination was observed since a significant reduction of viral load in serum compared to the control group was detected in all sampling days after challenge; efficacy was supported by the significant reduction of nasal and oral shedding as well as in rectal temperatures. Clinical signs were not expected after the inoculation of a PRRSV-1 subtype 1 challenge strain. However, the challenge virus was able to develop fever in 61% of the control pigs. Vaccination had a positive impact on rectal temperatures since the percentage of pigs that had fever at least once after challenge was reduced to 31% in vaccinated animals, and control pigs had significantly higher rectal temperatures than vaccinated pigs 3 days post-challenge. The lack of a vaccination effect in body weight gain was probably due to the short evaluation period after challenge (10 days). In the vaccinated group, 9/16 pigs (56%) experienced an increase in ELISA S/P ratio from the day of vaccination to 67 days post-vaccination. All vaccinated pigs were seropositive before challenge, indicating the development of an antibody response following vaccination even in the face of MDAs. In contrast to ELISA results, only 2/16 vaccinated pigs developed neutralizing antibodies detectable by a SNT that used a subtype 1 MA-104 adapted strain. Even in the absence of SN antibodies, vaccinated pigs were protected from challenge with a heterologous strain. The role of cell-mediated immunity should be considered, if protection was not mediated by SN antibodies only.

**Conclusions:**

The efficacy of the attenuated PRRSV-1 vaccine in 1-day-old pigs seropositive to PRRSV prior to a PRRSV-1 challenge was demonstrated by improvement of clinical, virological and immunological variables. With the current experimental design, maternal immunity did not interfere with the development of a protective immune response against a PRRSV-1 challenge, after vaccination of 1 day-old pigs. Confirmation of these results under field conditions will be needed.

## Background

Porcine Reproductive and Respiratory Syndrome Virus (PRRSV) is the causative agent of a disease that affects pigs worldwide and produces large economic losses to the swine industry [1]. It belongs to the genus Arteriviridae and two different species are now recognized: PRRSV-1 (formerly genotype 1), grouping European isolates, and PRRSV-2 (formerly genotype 2), grouping North American and Asian isolates [2]. The disease is characterized by reproductive disorders in sows and respiratory disorders in pigs. Weaner and grower pigs show mainly varying degrees of respiratory distress, and up to 20% of pigs may die. The incidence of other infectious diseases is increased and these may include meningitis caused by *Streptococcus suis*, bacterial bronchopneumonia and Glässer’s disease [3, 4].

PRRSV usually becomes endemic in infected herds and clinical disease is then observed in highly susceptible groups like weaned pigs in which passive immunity has waned, or naïve pigs introduced into the herd such as gilts and young boars [5]. Having immunity in place when piglets are weaned can protect them from early infections; early infections are apparently increasing in recent years in some specific countries [6]. Due to the effect of maternally derived immunity in newborns, vaccination is usually delayed until 3–4 weeks of age. The duration of maternal derived antibodies (MDA) has been described to be in the range of 6 and 11 weeks [7, 8]. Consequently, many pigs are vaccinated while still having maternal immunity in place. Most of the vaccines commercialized in Europe have a specific warning regarding interference by maternal-derived antibodies; thus, the protection induced by these vaccines according to the current vaccination practice in piglets may be compromised.

Once pigs are vaccinated, the onset of immunity against PRRSV can take 3 to 4 weeks to develop [9, 10]. Moreover, in animals with high maternal antibody titers, the post-vaccination immune response may be hampered for at least 4 weeks [8]. Due to this, piglets can have a period of risk for PRRSV infection, in which maternal immunity is no longer acting, and vaccine immunity has not yet been developed.

In Europe, the interference of maternal immunity with vaccine efficacy has been demonstrated at both the immunological and virological levels [8, 11, 12]. In a study conducted in France, it was demonstrated that pigs vaccinated when maternal antibody titers were high presented a delayed development of vaccine-related immunity, measured by both total antibody titers and neutralizing antibody titers [8]. However, whether this impairment of vaccine-related immunity development resulted in a lack of protection against exposure to a wild type virus was not investigated. In a second study conducted in France, it was demonstrated that pigs vaccinated when maternal antibody titers were high had a lower percentage of seroconversion, and, after challenge, viremia was not reduced, compared to non-vaccinated and challenged animals [12]. However, the level of neutralizing antibodies in non-vaccinated animals at challenge was not known, and if they had not declined there is the possibility that the challenge did not take and the absence of differences was due to this fact. In a study conducted in Italy, it was demonstrated that pigs vaccinated when maternal antibody titers were high presented PRRSV viremia values that were similar to non-vaccinated pigs, when a wild type virus circulated in the farm [11]. Since this study evaluated the effect of PCV2 and PRRSV vaccination on the clinical outcome of field exposure to multiple infectious agents at farm level, it was not possible to determine if clinical differences attributable to PRRSV infection were observed between vaccinated and control pigs. In Korea, lack of interference of maternal immunity with early vaccination has been demonstrated, using a MLV vaccine based in a PRRSV-2 strain, at the clinical and immunological level, but not at the virological level [6]. Thus, additional studies are needed to characterize the potential interference of maternal immunity with attenuated PRRS vaccines.

Early vaccination of piglets, when lack of interference by maternal immunity can be demonstrated, can be used in those situations in which early circulation of PRRSV occurs after weaning. The usefulness and lack of interference of 1-day-old piglet vaccination has already been demonstrated with a PRRSV-2 based modified life virus (MLV) vaccine [6]. The objective of the present study was to investigate the potential interference of vaccination of pigs from 1 day of age with a commercial PRRSV-1 based attenuated vaccine (Suvaxyn PRRS MLV) in presence of maternal immunity. Immunological, virological and clinical parameters were used to evaluate the outcome of vaccination in an experimental challenge model.

## Methods

### Experimental design

To produce PRRSV MDA positive piglets, six pregnant seronegative sows (coming from a PRRSV naïve farm) were vaccinated with a PRRSV-1 based attenuated vaccine (Suvaxyn PRRS MLV) at maximum release dose (10^5.2^ TCID_50_/dose) during the first half of gestation (45 days of pregnancy). The day before the expected farrowing date, parturition was induced with an intramuscular injection of cloprostenol (Cyclix® Porcino, Virbac). All sows farrowed the next day.

Thirty-four one day-old piglets born from PRRSV-seropositive sows were used. Before farrowing, sows were randomly assigned to two rooms. Treatments were randomly assigned to sows within rooms using a completely randomized design. Immediately after birth and prior to vaccination, piglets were cross-fostered such that piglets were randomized and spread as even as possible over all sows. At weaning sows were removed and piglets were moved into three rooms. Cross-fostered litters were randomly assigned to rooms and crates such that all animals from the same treatment were housed in the same room. Prior to challenge, animals were comingled within four pens such that original litters were kept together and all treatments were represented within each pen.

At 1 day of age, 16 pigs were administered a single 2 mL dose of vaccine via the intramuscular route (T02). Eighteen pigs from the control group (T01) received 2 mL intramuscular and 2 mL intranasal of saline solution. At the age when the MDA levels detected by serum neutralization test (SNT) in the T01 group were negative (SNT titer ≤1:2) all pigs were challenged with PRRSV Olot/91 and at 10 days later they were euthanized and necropsied (Table 1). PRRSV viral load in serum, lung lesions, rectal temperatures, nasal and oral shedding, clinical signs and body weight were evaluated.

**Table 1:** Experimental design

Group	Treatment	Dose	N° pigs	Day of vaccination	Day of challenge (DC)	Sampling days	Necropsy
T01	Saline solution	2 mL IM + 2 mL IN	18	D0 (1 day-old)	D67 (10-week-old)	D70, D73, D75, D77 (DC + 3, DC + 6, DC + 8, DC + 10)	D77 (DC + 10)
T02	Suvaxyn PRRS MLV	2.1 log_10_ CCID_50_/2 mL	16				

### Vaccination

Piglets of 24 ± 12 h were used. A PRRSV-1 based attenuated vaccine (Suvaxyn PRRS MLV) was used for T02, below the minimum immunizing dose (10^2.1^ TCID_50_/dose). At day 0, piglets of T02 were injected intramuscularly in the right side of the neck. Piglets of T01 received 2 mL intramuscular and 2 mL intranasal of saline solution.

### Challenge

At 67 days post-vaccination all pigs were challenged intranasally with the PRRSV-1 subtype 1 isolate Olot/91 [13], at a dose of 10^4.3^ CCID_50_/pig. The challenge virus was a passage 8 in PAM and shared only 90.6% genomic nucleotide identity with the vaccine strain.

### Sampling

Sows were bled at farrowing and sera were tested by ELISA and by SNT to a subtype 1 field strain, to confirm the seropositive status of sows to PRRSV.

Before vaccination, at day 0, piglets were bled to be tested by SNT to the vaccine strain, with the aim to detect MDA interference with vaccination.

After vaccination, control pigs were bled at day 52, to be tested by SNT to the vaccine strain, with the aim to determine the MDA decay and establish the day of challenge.

All pigs were bled just before challenge and sera tested by SNT to a MA-104 adapted subtype 1 strain, with the aim to determine the presence of NA that could be directed to the challenge strain. The challenge strain itself could not be used in this assay due to the fact that, in the testing laboratory, is not adapted to MA-104.

Before challenge (day 67) and after challenge, all pigs were bled and nasal and oral swabs were taken at days 3, 6, 8 and 10 (study days 70, 73, 75 and 77).

Blood was collected in the adequate containers to obtain serum. Nasal and oral swabs were placed in 1 mL of PBS. Samples were tested by PRRSV RT-qPCR to quantify PRRSV load.

### Clinical observations and body weight

At the same days of sampling, clinical observations including general condition, depression, sneezing, coughing, respiratory distress and others (if present) were made. Rectal temperatures were also taken those days. Body weight was measured at birth, the day of challenge and the day of necropsy.

### Macroscopic lung lesion scoring

After euthanasia, lungs were extracted from the thoracic cavity. Lung macroscopic lesions were immediately scored using the following method: the percentage of consolidation for each lobe (left cranial, left middle, left caudal, right cranial, right middle, right caudal and accessory) was scored and recorded as percent of lobe observed with lesions. Percentage of total lung with lesions was calculated using the following formula: Percentage of total lung with lesions = (0.10 x left cranial) + (0.10 x left middle) + (0.25 x left caudal) + (0.10 x right cranial) + (0.10 x right middle) + (0.25 x right caudal) + (0.10 x accessory).

### PRRSV ELISA test

Sow sera at farrowing, and piglet sera collected before vaccination (D0), before challenge (D67) and at necropsy (D77) was tested for antibodies to PRRSV using a PRRSV ELISA test (IDEXX PRRS X3), following the manufacturer’s instructions.

### PRRSV serum neutralization assays

Both SNT described below have been adapted from a previously described method [14].

SNT to vaccine strain: Heat inactivated serum samples were two-fold diluted (1:2 to 1:4096) in 96-well plates. One-hundred CCID_50 _of the vaccine strain 96 V198 were added to each well and plates were incubated for 1 h at 36–38 °C. A BHK21-CD163 expressing cell suspension containing 2.5-3 × 10^5^ cells/mL was prepared and 100 μL were added to each well in a new plate. Fifty microliters of the sample + virus mix was transferred to the plate containing cells. The mixture was incubated at 37 °C ± 1 °C and 5% CO_2_ for five days. A direct immunofluorescence assay technique using SDOW-17 FITC conjugated as primary antibody was performed. The neutralizing antibody (NA) titer was determined as the inverse of the last dilution of serum that inhibited the immunofluorescence signal.

SNT to MA-104 adapted subtype 1: Heat inactivated serum samples were two-fold diluted (1:2 to 1:4096) in 96-well plates. One hundred CCID_50_ of the subtype 1 strain were added to each well and plates were incubated overnight (18–24 h) at 2–8 °C. An MA-104 cell suspension containing 2.5-3 × 10^5^ cells/mL was prepared and 100 μL were added to each well. The mixture was incubated at 37 °C ± 1 °C and 5% CO_2_ for one week. The NA titer was determined as the inverse of the last dilution of serum that inhibited the cytopathic effect.

### PRRSV RT-qPCR

RNA was purified from serum and nasal swab samples using a commercial kit and a semi-automatic system (Biosprint 96 DNA Blood kit). Viremia was measured by means of a Reverse Transcription (RT) qPCR. In brief, viral RNA was purified from 200 μl of sample. Elution was performed in 100 μl. Five μl of RNA were used as template, reverse transcribed at 50 °C for 30 min, and denatured at 95 °C for 5 min. The PCR program consisted of 40 cycles of denaturation at 95 °C for 20 s and annealing/extension at 53 °C for 40 s. The RT-qPCR was conducted in a thermocycler (7500 Real-Time PCR System).

The sequences of primers and probe were the following:

- Forward primer (Lelystad F): 5’-GCACCACCTCACCCAGAC-3′ (Final concentration 0.5 μM).
- Reverse primer (Lelystad R): 5’-CAGTTCCTGCGCCTTGAT-3′ (Final concentration 0.5 μM).
- Probe (Lelystad S): 5′-6-FAM- CCTCTGCTTGCAATCGATCCAGAC –TAMRA-3′ (Final concentration 0.6 μM).

To quantify the viral load, the number of RNA copies obtained per 5 μl of reaction were × 100 and the result was expressed as number of RNA copies/mL of sample.

### Data analysis

Data summaries and analyses of data were performed with a centralized data management system (SAS/STAT User’s Guide Version 9.3 or higher, SAS Institute, Cary, NC). Only post challenge data (once animals were comingled) were analyzed. Pre-challenge data was summarized with descriptive statistics. Prior to statistical analysis results were transformed, where necessary, using an appropriate logarithm transformation. For viral load analysis, negative samples were given a value of 1.7 log_10_ RNA copies/mL, which is half the limit of quantification of the technique.

Viral load, serology, body weight, and rectal temperature were analyzed with a generalized linear repeated measures mixed model with fixed effects: treatment, time point, and treatment by time point interaction, and random effects: pen, block within pen, and animal within block, pen, and treatment, which is the animal term. Linear combinations of the parameter estimates were used in a priori contrasts after testing for a significant (*P* ≤ 0.05) treatment effect or treatment by time point interaction. Comparisons were made between treatments at each time point. The 5% level of significance (P ≤ 0.05) was used to assess statistical differences. Least squares means (back transformed for viral load and serology), standard errors, 95% confidence intervals of the means and ranges were calculated for each treatment and time point.

The percent of positive piglets was analyzed with a general linear mixed model with fixed effect treatment and random effect pen and block within pen. Pair-wise treatment comparisons were made if the treatment main effect was significant (*P* ≤ 0.05). Fisher’s Exact test was used for analysis if the mixed model did not converge.

The arcsine square root transformation was applied to the percentage of total lung with lesions prior to analysis. Transformed percentage of total lung with lesions was analyzed with a general linear mixed model with fixed effects, treatment, and random effects pen and block within pen. Linear combinations of the parameter estimates were used in a priori contrasts after testing for a significant (*P* ≤ 0.05) treatment effect. Comparisons were made between treatments. The 5% level of significance (P ≤ 0.05) was used to assess statistical differences. Least squares means (back-transformed), standard errors, 95% confidence intervals of the means and ranges were calculated for each treatment.

All hypothesis tests were conducted at the 0.05 level of significance using two-sided tests.

## Results

### Sow serology

All sows were seropositive to PRRSV at farrowing, with ELISA S/P ratios ranging between 0.887 and 2.204 and NA titers ranging from < 1:2 to 1:8 (Table 2).

**Table 2:** Sow serology at farrowing (ELISA and NA titers)

Sow ID	ELISA S/P ratio	Inverse NA titer
302	1.140	< 2
517	1.222	NT
336	2.023	3
343	0.887	3
513	2.204	8
511	0.955	4

ELISA positive: S/P ratio ≥ 0.4; SNT positive ≥2; *NT*: not tested

### Piglet serology (SNT)

Sera collected from control pigs at day 52 was used to determine if MDA had decayed enough to proceed to challenge. All sera tested negative (< 1:2) to a MA-104 adapted subtype 1 strain. Based on these results, it was considered that the levels of MDA in the control T01 group were low enough to ensure a successful challenge take.

**Fig. 1:**
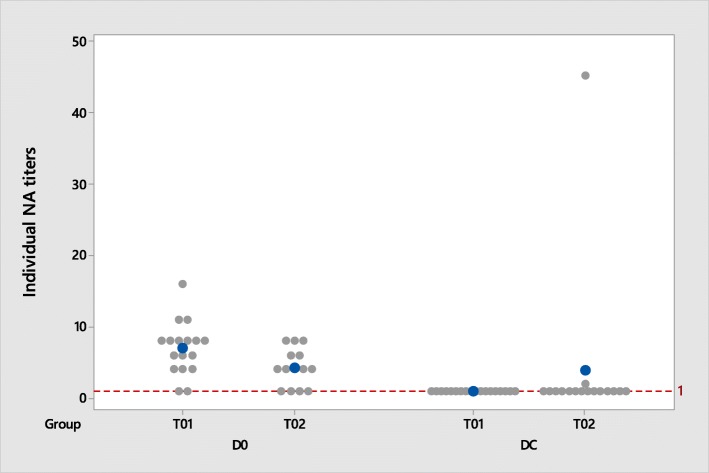
Neutralizing antibody titers to vaccine strain (D0, day of vaccination) and to field-type heterologous strain (DC, day of challenge); positive > 1,0

Sera collected from all pigs at day 0 and 67 were evaluated for the presence of PRRSV-specific neutralizing antibodies by means of an SNT to the vaccine strain (day 0) and to a MA-104 adapted subtype 1 strain (day 67) (Fig. 1).

Just before vaccination, once all piglets had suckled colostrum, NA titers were ranging from < 1:2 to 1:11 in control pigs (15 out of 18 pigs with NA titers ≥1:2) and < 1:2 to 1:8 in vaccinated pigs (10 out of 16 pigs with NA titers ≥1:2).

Just before challenge, NA titers were negative (< 1:2) in all control pigs and were ranging from < 1:2 to 1:45 in vaccinated pigs (2 out of 16 pigs with NA titers of 1:2 and 1:45).

### Piglet serology (ELISA)

**Fig. 2:**
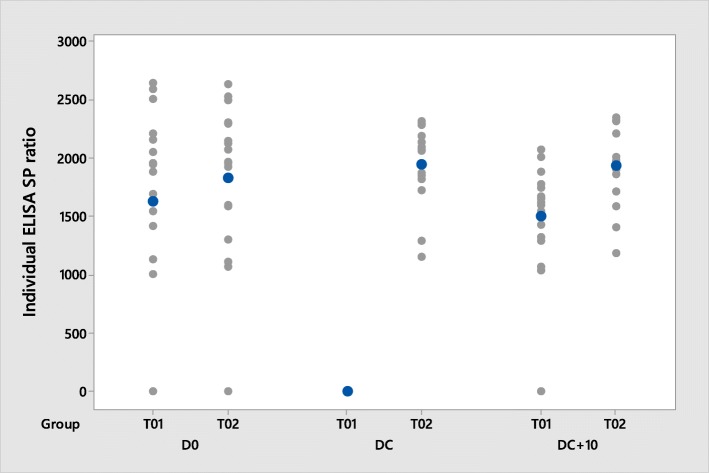
Mean ELISA S/P ratio at vaccination (D0), at challenge (DC) and at necropsy (DC + 10); ELISA positive: S/P ratio ≥ 0,4

All pigs had presence of PRRSV-specific MDA prior to vaccination as detected by ELISA (S/P ratio ≥ 0.4). The mean S/P ratio was 1.662 in the control group and 1.836 in the vaccinated group (Table 3, Fig. 2).

**Table 3:** Summary of ELISA results (S/P ratio) in piglets

Treatment Number	Day of Study	Geometric Mean/LSM^a^	SE	Range	% of seropositive
T01	Day 0	1.662	0.437	0.609 to 2.641	100.0
T02	Day 0	1.836	0.358	0.967 to 2.632	100.0
T01	Day 67	0.279	0.015	0.021 to 0.747	38.9
T02	Day 67	1.803	0.101	1.151 to 2.310	100.0
T01	Day 77	1.517	0.111	0.543 to 2.070	100.0
T02	Day 77	1.794	0.096	1.187 to 2.343	100.0

^a^Day 0 results are expressed with the Geometric Mean and Days 67 and 73 with the Back transformed – Least Square Mean (LSM); SE: standard error; ELISA positive: S/P ratio ≥ 0.4

At challenge (D67), all (100%) vaccinated pigs (T02) were seropositive to PRRSV by ELISA. In the control group (T01), 7/18 pigs (39%) had also detectable PRRSV antibodies before challenge. The mean S/P ratio was 0.279 in the control group and 1.803 in the vaccinated group.

Ten days after challenge (D77), all pigs were seropositive to PRRSV.

The levels of PRRS antibodies detected before challenge (67 days post-vaccination) in the vaccinated group were significantly higher (*p* < 0.0001) compared to the levels detected in the control group.

### Clinical observations and body weight

No clinical observations (general condition, depression, respiratory distress, cough, sneeze or other) were recorded for any pig during the whole observation period.

Before challenge, the least squares mean rectal temperatures was significantly lower in T02 group compared to T01. However, none of the pigs from any group had fever (rectal temperature ≥ 40.5 °C) at that time. After challenge, the percentage of pigs that had fever at least once was 61%, and 31%, in control and vaccinated groups, respectively. Comparison between groups showed that pigs from the control T01 group had significantly higher (*p* = 0.0005) rectal temperature than pigs from T02 group at day 70 (3 days post-challenge).

Regarding body weight, the mean for T01 at birth was 1.4 kg and for T02 was 1.3 kg. Comparison of least squares means between groups at D67 (challenge) and D77 (necropsy) showed no significant differences between groups, although the least squares mean starting weight at challenge was 28.7 kg for T01 and 28.1 kg for T02, and the least squares mean weight at necropsy was 31.0 for T01 and 32.0 for T02.

### Viremia

All pigs were found RT-qPCR PRRSV negative in serum before vaccination (D0) and all pigs from the T01 group remained so until challenge. In contrast, 8/16 (50%) piglets from the T02 group were RT-qPCR PRRSV positive at challenge (67 days post-vaccination).

After challenge, 100% of pigs from the T01 group became viremic at D70 (3 days post-challenge) and remained positive until the end of the study at 10 days post-challenge. In the vaccinated group T02 all pigs were detected PRRSV positive at least once; however, by the end of the study (DC + 10), only 11/16 T02 pigs (68.8%) were still viremic (Table 4).

**Table 4:** Least squares mean (±standard error) viral load in serum and nasal/oral swabs, and percentage of RT-qPCR positive pigs (in brackets). Results from RT-qPCR are expressed as log_10_ RNA copies/mL of serum. A positive result is considered when > 1.7 log_10_ RNA copies/mL (limit of quantification)

	Treatment	Day of study					
		D0	D67 (Ch)	D70 (Ch + 3)	D73 (Ch + 6)	D75 (Ch + 8)	D77 (Ch + 10)
Viremia	T01	≤ 1.7 (0%)	1.65 ± 0.20 (0%)	6.60 ± 0.20 (100%)	6.39 ± 0.20 (100%)	5.32 ± 0.20 (100%)	5.29 ± 0.20 (100%)
	T02	≤ 1.7 (0%)	2.25 ± 0.36 (50%)	2.87 ± 0.36 (50%)	5.18 ± 0.36 (94%)	4.18 ± 0.36 (100%)	2.96 ± 0.36 (69%)
	T01 vs T02 (*p* value)	NT	0.1495	< 0.0001	0.0043	0.0071	< 0.0001
Nasal shedding	T01	NT	≤ 1.7 ± 0.19 (0%)	3.91 ± 0.19 (100%)	3.98 ± 0.19 (100%)	2.27 ± 0.19 (61%)	1.90 ± 0.19 (22%)
	T02	NT	≤ 1.7 ± 0.20 (0%)	1.81 ± 0.20 (12%)	2.72 ± 0.20 (50%)	2.35 ± 0.20 (44%)	1.70 ± 0.20 (0%)
	T01 vs T02 (*p* value)	NT	0.9986	< 0.0001	< 0.0001	0.7576	0.4654
Oral shedding	T01	NT	1.70 ± 0.03 (0%)	3.34 ± 0.21 (83%)	3.68 ± 0.23 (94%)	2.57 ± 0.19 (67%)	2.28 ± 0.20 (39%)
	T02	NT	1.75 ± 0.03 (6%)	2.32 ± 0.22 (56%)	3.62 ± 0.25 (81%)	2.30 ± 0.20 (50%)	1.80 ± 0.22 (12%)
	T01 vs T02 (*p* value)	NT	0.1978	0.0009	0.8800	0.3243	0.1096

*NT*: not tested

**Fig. 3:**
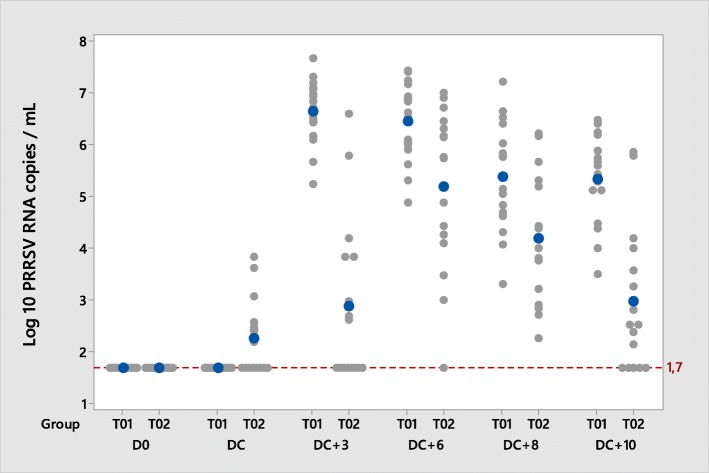
PRRSV viral load in serum during the post-challenge phase; PRRSV RT-qPCR positive > 1.7 PRRSV RNA copies/mL

Pigs from the T02 group had significantly lower viral load in serum than pigs from the T01 control group at all sampling days post-challenge (Table 4, Fig. 3).

### Nasal and oral shedding

All pigs were found RT-qPCR PRRSV negative in nasal swabs before challenge, and all but one (belonging to T02 group) were found RT-qPCR PRRSV negative in oral swabs.

After challenge, the percentage of pigs that ever shed PRRSV by nasal route in the T01 group was significantly higher compared to T02 (100% vs.75%) (Table 4). All pigs (T01 and T02) became oral shedders, except one pig from T02 group that was negative at all sampling points post-challenge.

**Fig. 4:**
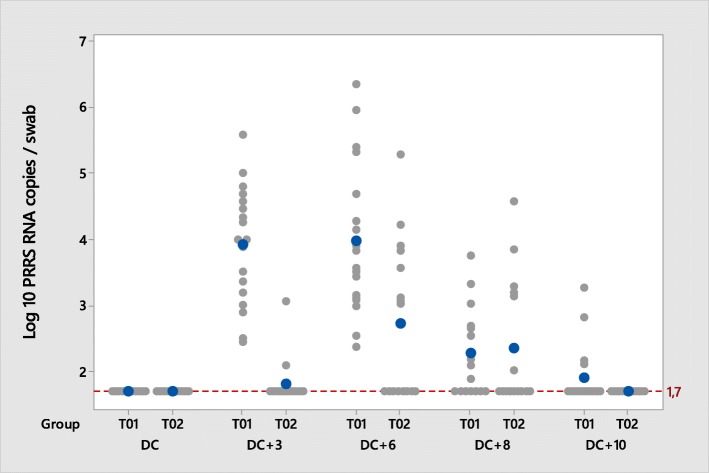
PRRSV viral load in nasal swabs during the post-challenge phase; PRRSV RT-qPCR positive > 1.7 PRRSV RNA copies/mL

**Fig. 5:**
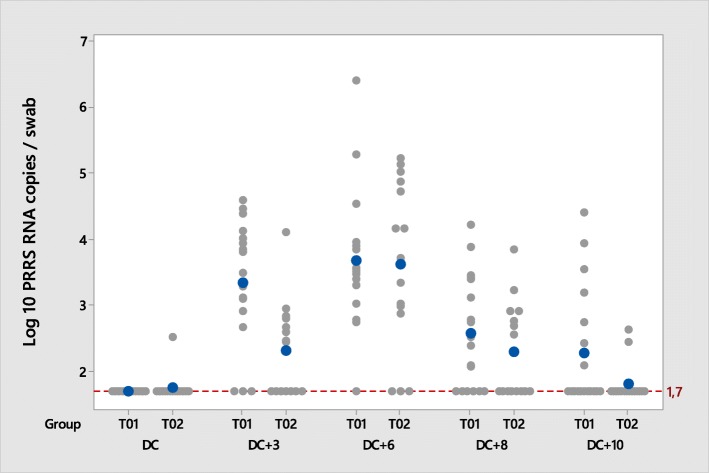
PRRSV viral load in oral swabs during the post-challenge phase; PRRSV RT-qPCR positive > 1.7 PRRSV RNA copies/mL

The amount of virus shed by the nasal and oral routes was significantly lower in the T02 group compared to T01 group at day 70, corresponding to 3 days post-challenge (Figs. 4 and 5). At day 73 (6 days post-challenge) the amount of virus shed by the nasal route was significantly lower in the T02 group compared to T01 (Table 4).

### Macroscopic lung lesions

**Fig. 6:**
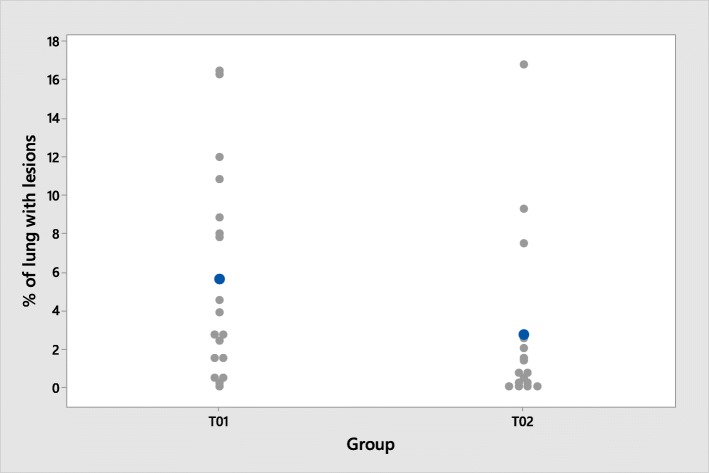
Distribution of the individual lung macroscopic lesion scores

At necropsy, 13/18 pigs (72%) from the control group T01 had a positive lung visual score, indicating that PRRSV challenge was successful in inducing lung lesions. In the T02 group, 7/16 (44%) pigs scored positive as well (Fig. 6).

Comparison between treatment groups showed no significant differences (*p* = 0.092) in the % of lung with lesions (4.3% in control pigs vs 1.3% in vaccinated pigs).

## Discussion

Early vaccination of piglets against PRRSV, when non-interference with passively acquired immunity can be demonstrated, is a useful tool to control PRRSV-related disease in young animals. The objective of the present study was to evaluate the effect of maternally derived immunity on the efficacy of a PRRSV-1 based MLV, when administered in 1 day-old piglets by the intramuscular route. Efficacy was evaluated in seropositive pigs vaccinated at 1 day of age upon inoculation with a pathogenic PRRSV-1 isolate, heterologous to the vaccine strain, as a respiratory challenge. The animals were challenged when the levels of MDAs detected by SNT in the control group became undetectable, to guarantee challenge take in control animals and the ability to detect differences between the treatment groups.

Historically PRRSV-1 subtype 1 isolates, the most predominant in Western Europe, have showed a very limited ability to induce respiratory clinical signs compared to PRRSV-2 and subtypes 2 and 3 of PRRSV-1. However, some recent isolates (e.g. from Italy, Belgium, and Austria) may indicate a trend towards increasing virulence of subtype 1 [15–17]. The challenge strain used in this study, Olot/91, has been shown to be very aggressive when used in a reproductive model [13] but induces a mild disease in young pigs [18]. Consequently, other variables must be selected as primary variables to evaluate the outcome of infection. Viremia is the most frequently used parameter to verify PRRSV infection outcome in pigs [7, 19]. In the present study, a protective effect of vaccination was observed when comparing viral load in serum between the vaccinated and control groups. The vaccinated group had significantly lower viral titers compared to the control group at all sampling days post-challenge. The vaccine strain was still detected at low levels in some vaccinated animals before challenge; consequently, the amount of virus detected in this group in the following days was probably a mixture of vaccine strain and challenge strain. Since the values obtained were analyzed as being all due to challenge, the results could not favor the interpretation of vaccine efficacy.

The protection conferred following vaccination was supported by the significant reduction in the percentage of nasal shedders as well as in the amount of virus detected in nasal and oral secretions in the vaccinated group in relation to the control group. These results are in contrast of those recently reported [12]; in that case, a clear interference of maternally derived neutralizing antibodies with PRRSV vaccination was described (no significant differences in viremia reduction and lower transmission rate estimated for pigs vaccinated with low antibody titers than for pigs vaccinated with high antibody titers).

It has been described that the ability of PRRSV attenuated vaccines to control the disease (measured by reduction of viremia) appears to be much lower in the field than under experimental conditions [11, 20]. In these studies, the same PRRSV attenuated vaccine performed differently when used under laboratory conditions (significant reduction of the magnitude and duration of viremia) and under field conditions (no significant reduction of viremia). Although it has been suggested that this could be linked to the influence of maternal derived antibodies, this could not be used as a universal explanation, since in some cases this effect has been observed in piglets that were seronegative at vaccination [21]. Moreover, when 1-day-old piglets were vaccinated in the presence of maternal antibodies [6], a very limited effect in reduction of wild type virus viremia was observed. However, the efficacy of vaccination was demonstrated by improved growth performance and reduced mortality. Thus, any demonstration of PRRSV vaccination interference by maternal antibodies should be verified under both laboratory and field conditions.

Clinical signs, mainly respiratory signs, were not expected after the inoculation of the challenge strain Olot/91, as described in previous reports [18]. However, the challenge virus was able to induce fever in 61% of the control pigs. Vaccination had a positive impact on rectal temperatures since the percentage of pigs that had fever at least once after challenge was reduced to 31% in vaccinated animals, and control pigs had significantly higher rectal temperatures than vaccinated pigs 3 days post-challenge. The lack of a positive vaccination effect in body weight gain was probably due to the short evaluation period after challenge (10 days). Those reports demonstrating improved daily weight gain after vaccination have considered much wider periods of analysis [6, 11, 21]. The difference in body weight between birth and challenge was not statistically analyzed because during the post-vaccination phase the treatment groups were not commingled. The difference of 0.6 kg between vaccinated and control groups (in favor of the control group) was overcome by the vaccinated group after challenge.

At necropsy, 13/18 pigs (72%) from the control group had developed macroscopic lung lesions compatible with PRRSV infection. In contrast, only 7/16 (44%) vaccinated pigs developed lesions. Comparison between treatment groups showed no significant differences in the % of lung with lesions. However, the differences observed were close to significance (*p* = 0.092), indicating that these differences might have a biological relevance. Taken together the clinical, virological and pathological data clearly indicate that vaccinated animals were able to better respond to PRRSV infection than non-vaccinated animals.

All pigs had PRRSV-specific antibodies before vaccination as measured by ELISA (S/P ratio ≥ 0.4), thus complying with the inclusion criteria. Before challenge (67 days post-vaccination), 39% of the pigs in the control group were still seropositive, indicating the presence of remaining MDAs at that time (mean S/P ratio: 0.279). However, the fact that all pigs from the control group developed viremia after challenge and that 13/18 had also a positive lung score at necropsy indicates that the remaining MDA detected by ELISA did not interfere with the challenge take. In fact, when the levels of PRRSV-specific NA were determined in those pigs by means of an SNT, all T01 pigs were below the level of detection before challenge (Day 52).

In the vaccinated group, 9/16 pigs (56%) experienced an increase in the ELISA S/P ratio from the day of vaccination to 67 days post-vaccination and all of them were seropositive before challenge (mean S/P ratio: 1.803), indicating the development of an antibody response following vaccination even in the face of MDAs. These results are in agreement with those recently reported [12], in which 44% of piglets vaccinated in presence of maternal immunity seroconverted 4 weeks later. In contrast to ELISA results, only 2/16 vaccinated pigs developed serum NA detectable by a SNT that used a subtype 1 field strain. Since the strain used in the SNT was not the challenge strain (although both were subtype 1 strains), the low number of pigs having NA could be due to the previously described effect of the use of a heterologous strain in the assay [14, 22]; if that was the case, NA could have been under-detected. Even in the absence of NA antibodies, vaccinated pigs were partially protected from challenge with a heterologous strain (as demonstrated by virological and clinical variables). The role of cell-mediated immunity should be considered, if protection was not mediated by NA only. It has been suggested that protection against PRRSV infection is not based on humoral immunity only, and that a combination of NA and virus-specific IFN-γ secreting cells is needed to achieve clearance of PRRSV infection [23].

The NA titers detected before vaccination were considered moderate to low, below the demonstrated level of interference in seroconversion [8] and below the proposed limit of protection [19], which has been set at 1:8. However, they were generated using the most stringent conditions possible: vaccination of sows with the maximum antigen titer according to label (to achieve maximum level of MDAs in the newborn piglets). To achieve higher titers before vaccination, repeated vaccination of sows should be considered for future studies. The combination of sow vaccination with maximum antigen titer and piglet vaccination with the same strain as sows, homologous to the one that elicited the MDAs, represents the worse-case scenario for demonstrating the potential for maternal immunity interference with vaccination in terms of affinity of NA with the vaccine strain. However, in field conditions the scenario may be even more challenging for overcoming immunity, when sows are repeatedly vaccinated and exposed to different field strains.

Although both vaccine and challenge strains are PRRSV-1 subtype 1, they are far from being homologous, since they share 90.6% of nucleotide identity only. Studies with more divergent strains would be needed to confirm the results of the present study.

The reasons the outcomes of the current study are different from a similar study carried out in France [12] are not known. There are obvious differences in study design that might affect the outcomes of the two studies. The age of the pigs at vaccination, the vaccine used, and the timing of challenge administration are important factors that may have influenced the results. On the other hand, the results obtained in the current study are in agreement with those reported for a similar vaccine based on a PRRSV-2 strain, which demonstrated that vaccination may overcome maternal immunity yielding an improvement of growth performance in 1 day-old vaccinated pigs [6].

## Conclusions

The efficacy of an attenuated PRRSV-1 vaccine (Suvaxyn PRRS MLV) in 1-day-old seropositive pigs was demonstrated by an improvement in clinical, virological and immunological variables. Thus, with the current experimental design, maternal immunity did not interfere with the development of a partially protective immune response against a PRRSV-1 challenge, after vaccination of 1 day-old pigs. Confirmation of these results under field conditions will be needed.

## Abbreviations

CCID_50_: Cell Culture Infectious Dose 50%
ELISA: Enzyme linked immunosorbent assay
FITC: Fluorescein isothiocyanate
IFN-γ: Interferon gamma
MDA: Maternally Derived Antibodies
MLV: Modified Live Vaccine
NT: Not tested
PBS: Phosphate Buffered Saline
PCV2: Porcine Circovirus Type 2
PRRSV: Porcine Reproductive and Respiratory Syndrome Virus
RNA: Ribonucleic acid
RT-qPCR: Quantitative Reverse-Transcription Polymerase Chain Reaction
S/P: Sample to Positive
SNT: Serum Neutralization Test
TMB: 3,3′,5,5’-Tetramethylbenzidine
